# Postbiotics in Human Health: A Narrative Review

**DOI:** 10.3390/nu15020291

**Published:** 2023-01-06

**Authors:** Linxi Ma, Huaijun Tu, Tingtao Chen

**Affiliations:** 1 Departments of Geriatrics, The Second Affiliated Hospital of Nanchang University, Nanchang 330031, China; 2 Queen Mary School, Nanchang University, Nanchang 330031, China; 3 National Engineering Research Center for Bioengineering Drugs and Technologies, Institute of Translational Medicine, Nanchang University, Nanchang 330031, China

**Keywords:** postbiotics, probiotics, comprehensive health, immunomodulation

## Abstract

In the 21st century, compressive health and functional foods are advocated by increasingly more people in order to eliminate sub-health conditions. Probiotics and postbiotics have gradually become the focus of scientific and nutrition communities. With the maturity and wide application of probiotics, the safety concerns and other disadvantages are non-negligible as we review here. As new-era products, postbiotics continue to have considerable potential as well as plentiful drawbacks to optimize. “Postbiotic” has been defined as a “preparation of inanimate microorganisms and/or their components that confers a health benefit on the host”. Here, the evolution of the concept “postbiotics” is reviewed. The underlying mechanisms of postbiotic action are discussed. Current insight suggests that postbiotics exert efficacy through protective modulation, fortifying the epithelial barrier and modulation of immune responses. Finally, we provide an overview of the comparative advantages and the current application in the food industry at pharmaceutical and biomedical levels.

## 1. Introduction

Previous decades have witnessed the rapid growth in productivity and industrialization, leading to consequent ecosystem quality challenges, such as air and water pollution, heavy metal pollution as well as mental stress resulting from a fast-paced life. Increasingly, more people complain about “sub-health” conditions, a state characterized by certain abnormalities in psychological or physical behaviors, or in some medical examination indicators with no typical pathologic symptoms [1] which has seriously threatened human physiological and psychological health. The concept of “comprehensive health” emerges to provide workable solutions to increase quality of life and life expectancy. A comprehensive health industry emphasizes disease prevention and health maintenance to avoid chronic diseases occurrence. In this regard, a rigid demand for functional food is increasing gradually.

Due to in-depth research on microorganisms, probiotics are widely applied in food processing, infant formula [2], medical, agriculture, and even in aquaculture industries [3] for their health-maintaining properties. Probiotics are referred to as dietary supplements that include live, nonpathogenic microorganisms which benefit the host’s health. They act via multiple mechanisms, involving immunomodulation, production of antimicrobial compounds, direct combination, or competitive inhibition of pathogens as well as regulation of electrolyte absorption and gut motility [4], etc. However, drawbacks such as quality fluctuations, short shelf life, heterogeneous effects and the user-unfriendliness of immunocompromised subjects limit its application during different transportation methods and storage conditions such as pasteurization or baking [5,6].

To solve this problem, new-era products such as postbiotics have emerged as the current research target, the properties of which are safer and more stable, easier to store and contain less risk of antimicrobial resistances. “Postbiotic” was defined as a “preparation of inanimate microorganisms and/or their components that confers a health benefit on the host” [5]. Typically, the forms could be a heterogeneous mixture of cellular structures and metabolites such as teichoic acids, exopolysaccharides, peptidoglycan, bacteriocins, etc. Three underlying mechanisms driving the efficacy of postbiotics include protective modulation against pathogens, enhancement of the epithelial barrier and the modulation of inflammatory and immune responses, respectively. At present, postbiotics are applied not only in the fermented food industry, but also as a promising treatment strategy for sub-health conditions [7], especially in gastrointestinal disorders including bloating and diarrhea. Therefore, the application of postbiotics would be an efficient complement to probiotics and a driving force for the development of a comprehensive health industry [8,9].

Given that postbiotics is a newly emerging concept, the lack of clear definitions and the ambiguous mechanisms remain to be optimized [10,11,12]. The purpose of this article is to review the main development process of the concept of “postbiotics” and the potential mechanisms. Meanwhile, through its dominant applications in the food industry and the pharmaceutical and biomedical fields, we summarize the advantages against the drawbacks and analyze the future development of postbiotics surrounding how to promote innovation and make an accurate market positioning. Above all, we hope to provide a theoretical basis and data support for probiotics application.

Based on our research on probiotics, prebiotics and flora disorder related diseases, we found multiple applications with huge potential for postbiotics as the extension direction of probiotics; therefore, we provide a review to summarize and compare the application of postbiotics. To prepare this review, different Databases (e.g., PubMed), search engines (e.g., GOOGLE SCHOLAR), and websites (e.g., CNKI) were used for the retrieval of articles, reviews and investigations. We retrieved papers published in the English or Chinese language with no time limitation, using the following keywords: postbiotic, probiotic, comprehensive health and gut microbiota.

## 2. The Narrow Applications of Probiotics Provide Favorable Circumstances for Postbiotics

Probiotics are generally defined as dietary supplements containing live, nonpathogenic microorganisms which improve the health condition of the host if administered in sufficient doses [13,14,15]. Probiotics and prebiotics have been extensively studied and applied in gut microbiota re-modulation [16]. *Bifidobacterium* and *Lactobacillus*, the predominant and subdominant groups among the gastrointestinal microbiota, are the most routinely utilized probiotic bacteria and are present in a wide range of health-beneficial products [17]. Probiotics have long been manufactured from other Gram-positive bacteria, namely those from *Streptococcus*, *Bacillus*, and *Enterococcus* genera. Additionally, it has been demonstrated that the *yeast Saccharomyces* offers health advantages, particularly in fermented dairy products [18]. *Bacteroides* and *Clostridium* genera have potential for the future despite some safety issues [19].

Probiotics are commonly applied in medical devices, infant formulas, fermented dairy products, nutritional supplements, and “biotic” feed additives [2,20]. Clinical potential has also been demonstrated through human studies and animal models, mainly gastrointestinal diseases involving lactose intolerance, irritable bowel syndrome (IBS), functional gastrointestinal disorders, or inflammatory bowel disease, but also extra-intestinal disorders such as hepatic encephalopathy [21,22]. Therapeutic potential such as attenuating carcinogenesis of gastrointestinal (GI) tract has also been found in probiotics such as Lactobacillus acidophilus CGMCC 878, for example [23]. Moreover, probiotics have gained widespread acceptance in pediatrics, in particular with positive outcomes in dealing with acute infectious diarrhea and preventing antibiotic-associated diarrhea [24,25,26]. Probiotics’ therapeutic benefits against rotavirus diarrhea and other pediatric atopic illnesses have also been reported and have provided encouraging data [26,27,28]. V. Anderhoof has published a succinct overview of potential future uses for probiotics in human health [29] which suggests it is worthwhile to create probiotics for managing inflammatory conditions, treating and avoiding allergy disorders, preventing cancer and diminishing the prevalence of respiratory diseases.

Probiotics exert efficacy through probiotic-pathogen interactions by adopting several defence mechanisms. They generate antimicrobial compounds, for instance, they secrete lactic acid to reduce pH or directly bind to Gram-negative bacteria to inhibit their growth [2,21]; therefore *lactobacilli* species are valuable to use as examples here [30,31]. The second mode of action is the competitive exclusion of pathogens [32]. To cause disease syndromes, pathogens must adhere to the gastrointestinal epithelium. Competitive inhibition occurs when probiotics compete for colonization space, nutrition and other growth factors on host mucosal surfaces [33], thus reducing the pathogens’ pathogenicity [34].

Probiotics have also been implicated in probiotic-host interactions through immunomodulatory capabilities. Numerous probiotic benefits are achieved through the equilibrium of pro- and anti-inflammatory cytokines. For instance, *lactobacillus* strains have been shown to limit the increase of human T-cells [35]. Probiotics enhance the epithelial barrier integrity after damage resulting from various pathological states, for instance, *Escherichia coli*-induced mucosal rupture [2,36]. Some strains can also strengthen the mucus barrier by triggering the production of mucin granules from Goblet cells, which prevents pathogen penetration [2]. Moreover, probiotics show synergistic effects with indigenous microflora in the prevention of enteropathogens [34].

**Figure 1:**
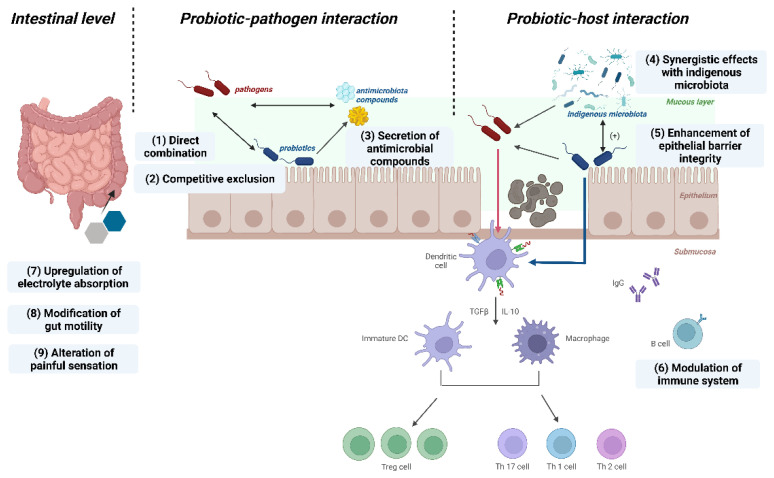
Predominant mechanisms of probiotic action. Probiotic-pathogen interactions in the middle part of the figure include three mechanisms: (1) direct combination, (2) competitive exclusion, (3) secretion of antimicrobial compounds; Probiotic-host interactions in the right part of the figure include three mechanisms: (4) synergistic effects with indigenous microbiota, (5) enhancement of epithelial barrier integrity, (6) modulation of immune system. At the intestinal level in the left part of the figure, probiotics have an effect through: (7) upregulation of electrolyte absorption, (8) modulation of gut motility, (9) alteration of painful sensations. (Figure was created with Biorender. com).

At the intestinal level, probiotics can alter gut motility and promote intestinal electrolyte absorption [21,37]. Probiotics can also affect how painful sensations are interpreted by modifying the expression of pain receptors and secreting potential neurotransmitter molecules, for example, by inhibiting the pain response to colorectal distension [34] (Figure 1).

In certain clinical populations, including immunocompromised patients, neonates, and vulnerable patients [38], the use of probiotics has sparked safety concerns. Probiotic microorganisms that are still in existence occasionally induce some of their own illnesses. These concerns mostly center on how microorganisms might transfer from the gastrointestinal system to the circulatory system and the cross-over of antibiotic resistance genes [39]. The studies demonstrate that probiotics used in clinics have undergone non-negligible quality fluctuations, more specifically, viability loss during subsequent processing steps such as centrifugation and drying [40]. Because of their original sensitivity to environmental changes, the ultimate products are more or less mixed with a fraction of inactivated cells [41]. Somewhat unpredictably, the quantity of dead cells may be even higher than that of living cells. The evaluation of the exact beneficial response would be influenced since the dosage was dependent on the number of living cells [34]. Additionally, probiotics may only temporarily colonize the digestive tract owing to the inevitable passage through the unsuitable environment in the stomach and small intestine [42,43]. Probiotics would be limited in environments with proteolytic enzymes or low pH, for example, *Streptococcus thermophilus* and *Lactobacillus delbrueckii* cannot withstand stomach acidity [34]. Interaction of probiotics included in the same formulations should also be taken into account because certain live probiotic species may diminish the stimulatory effects of others [44,45]. Equally notable is that the precise molecular mechanisms underlying a specific probiotic strain’s actions and the effects of various probiotic bacterial combinations at different immunological pathways remains unexplained [46]. Moreover, heterogeneous effects are commonly observed, which means that different strains may colonize the gut to different extents and elicit multiple immune responses despite sharing similar properties in vitro, making research and prescription more challenging [34].

## 3. Application of Postbiotics in Improving Human Health

### 3.1. Development History of the Concept “Postbiotics”

The concept that non-living microorganisms might improve or maintain health is not new, and several different terms have been used to describe these compounds (Table 1), although postbiotic has been the most commonly mentioned term in the last 10 years [5].

Scientific evidence that inactivated microorganisms have a favorable impact on human health has been steadily published in the literature since 2009 [47]. In 2011, scientists introduced the term “paraprobiotic” sometimes known as “ghost probiotics” to describe the application of inactivated microbial cells or cell fractions that when delivered in sufficient proportions, might offer a health benefit to the consumer [48]. Research in 2016 worked on paraprobiotic *Lactobacillus*, which clearly affected intestinal functionality due to the brain-gut interaction when continuously ingested [49]. Murata et al. conducted research on the effects of paraprobiotic *Lactobacillus* supplementation on common cold symptoms and mental states in 2018 [50]. Meanwhile, Deshpande reviewed current evidence indicating that paraprobiotics could be secure substitutes for probiotics in preterm infants by high-quality pre-clinical and clinical research [51]. Based on published clinical trial data up to 2018, Kanauchi described immune defense mechanisms and potential uses of paraprobiotics against viral infections [47]. The term “para-psychobiotic” also entered the field in 2017 in research exploring the effects of para-psychobiotic *Lactobacillus* on overstressed symptoms and sleep quality improvement [52].

Shenderov provided the first definition of “metabiotics” in 2013. Metabiotics are the structural components of probiotic microorganisms with or without their metabolites and signaling molecules which can optimize host-specific physiological functions, regulators, metabolic and behavior reactions associated with the indigenous microbiota [53]. Sharma’s clinical research from 2020 revealed that the isolated probiotic *Lactobacillus rhamnosus* produced metabiotics with antigenotoxic and cytotoxic properties against colon cancer [54]. The term “metabiotics” in the research mentioned above refers to a cell-free supernatant, which was collected by cold centrifuging overnight grown LAB cultures.

Heat treatments of bacterial suspensions can be manipulated in temperatures ranging from 70 to 100 °C. The method known as tyndallization, created by the scientist Dr. John Tyndall during the eighteenth century, allows for the inactivation of certain substances when heat treatments are combined with incubation periods at lower temperatures (ambient, cooling, or freezing temperatures) [55,56]. Probiotics are sterilized and suppressed to secrete active metabolites during the tyndallization process. The therapeutic benefits of “tyndallized probiotics” were verified in tyndallized *L. rhamnosus* for the first time for its therapeutic benefits on atopic dermatitis in 2016 [57]. In a study done in 2017, tyndallized probiotics are also discussed in the treatment of chronic diarrhea with gelatin tannate. Live probiotics that can generate active metabolites and tyndallized probiotics are two distinct forms of biological response modifiers, according to Lopetuso. Unable to reproduce, there is no risk for tyndallized probiotics to inherit antibiotic resistance genes or to induce sepsis [58]. Tyndallized probiotics and purified components exhibit probiotic capabilities, constituting a new generation of safer and more stable products, according to a review by Pique, et al. [4].

In 2018, Jurkiewicz et al. established the preventative effects of respiratory tract infections using “bacterial lysates” and combinations of numerous bacterial species which are responsible for respiratory tract inflammations to some extent [59].

**Table 1:** Evolvement of the concept “postbiotics”.

Concept	Date	Bacterial Strains Tested	Main Findings	Reference
Paraprobiotics or ghost probiotics	2009	Multiple	The health benefits of probiotics can be achieved without the risks related to administration of a live organism.	[47]
Paraprobiotics or ghost probiotics	2011	Multiple	Propose the new term “paraprobiotic” to refer to the inactivated microbial cells or cell fractions.	[48]
Paraprobiotics or ghost probiotics	2016	*Lactobacillus*	Paraprobiotic *Lactobacillus* affects intestinal functionality due to the brain-gut interaction.	[49]
Paraprobiotics or ghost probiotics	2018	*Lactobacillus*	*L. paracasei* MCC1849 improves resistance to common cold infections in vulnerable individuals and maintain a favorable emotional state.	[50]
Paraprobiotics or ghost probiotics	2018	Multiple	Paraprobiotics could be safe alternatives to probiotics in preterm neonates.	[51]
Paraprobiotics or ghost probiotics	2018	Multiple	Review the effectiveness of paraprobiotics for the prevention or treatment of virally-induced infections.	[60]
Para-psychobiotic	2017	*Lactobacillus*	Para-psychobiotic *Lactobacillus gasseri* CP2305 regulates stress responses depending on specific cell component(s).	[52]
Metabiotics	2013	Multiple	The concept, function and advantages of metabiotics.	[53]
Metabiotics	2020	*L. rhamnosus*	The isolated probiotic *L. rhamnosus* MD 14 generated metabiotics exhibiting antigenotoxic and cytotoxic effects against colon cancer.	[54]
Tyndallized probiotics	2016	*L. rhamnosus*	*L. rhamnosus* IDCC 3201 tyndallizate has potential for treating atopic dermatitis.	[57]
Tyndallized probiotics	2017	Multiple	Gelatin tannate and tyndallized probiotics can be used to restore the gut barrier physiological functions and prevent dysbiosis.	[58]
Tyndallized probiotics	2019	Multiple	Tyndallized bacteria and purified components confer probiotic properties.	[4]
bacterial lysates	2018	Multiple	Bacterial lysates minimize the incidence of recurrent respiratory infections in children and adults when orally administrated.	[59]

### 3.2. Mechanisms Driving Postbiotic Efficacy

#### 3.2.1. Protective Modulation against Pathogens

Although postbiotics influence microbiota temporarily, for the most part, they could indeed play a significant mechanistic role. According to in vivo research, molecules contained in postbiotics, namely lactic acid and bacteriocins, may have direct antimicrobial properties. For instance, organic acids belonging to lactic acid bacteria, bifidobacterial and other postbiotic strains primarily exert antimicrobial efficiency against Gram-negative pathogens, which has a dose-dependent effect [61]. The antibacterial action of the cell-free supernatants is thought to be mostly due to bacteriocins [62], for example, supernatants derived from the genera *Lactobacillus* and *Bifidobacterium* were also verified to have antibacterial properties against the invasion of enteroinvasive *E. coli* [16]. Different *Bifidobacterium* strains have produced bifidocins, which have a wide spectrum of bactericidal action against both Gram-positive and Gram-negative bacteria as well as certain yeasts. Additionally, when exposed to exopolysaccharides (EPS) isolated from *Bifidobacterium bifidum*, lactobacilli and other anaerobic bacteria grew more readily while enterobacteria, enterococci, or *Bacteroides fragilis* are inhabited [63]. The well-known antibacterial metabolite reuterin, which is generated by *Lactobacillus reuteri*, is assumed to function by oxidizing thiol groups in pathogenic gut bacteria [61,64]. Co-aggregation with *Helicobacter pylori* has reportedly been suggested as another potential underlying mechanism for such an action of lactobacilli-contained postbiotics products [65].

The biofilms of pathogenic bacteria are one of the serious hazards to the medical fraternity. Biofilm appears to be the primary cause of pathogenesis and treatment failure because of the antimicrobial resistance enclosed in the biofilm matrix [66]. Through inhibiting the production of biofilms and deconstructing already-formed biofilms, the pure teichoic acids isolated from *Lactobacillus* strains have exhibited inhibitory effects on biofilm formation of oral or enteric pathogens including *Streptococcus mutans*, *Staphylococcus aureus*, and *Enterococcus faecalis* [67,68,69]. Biosurfactants that are produced extracellularly or attached to cell walls have the amphiphilic feature, which helps deconstruct existing biofilms or prevents biofilm formation. Additionally, the features of wetting, foaming, and emulsification prevent bacteria from adhering to, establishing themselves in, and subsequently communicating in the biofilms [70].

Postbiotics can also competitively eliminate pathogens by competing for adhesion sites if the adhesions (e.g., fimbriae and lectins) in postbiotics remain normally functional after pretreatment. It is possible to view lectins extracted from or expressed by advantageous *lactobacilli* as prospective bioactive components for better prevention of gastrointestinal and urogenital infections. The isolated lectin domains of Llp1 and Llp2 not only exert inhibitory effects against the development of biofilms in a wide range of pathogens, involving uropathogenic *E. coli* and clinical *Salmonella* species, but they also interpose the adhesion of *L. rhamnosus* GG to gastrointestinal and vaginal epithelium [71]. *Lactobacillus acidophilus* in lyophilized and inactivated form massively increases *H. pylori* eradication rates when added to a regular anti-*H. pylori* eradication regimen, due to its powerful adherent ability to human intestinal absorptive and muco-secreting cells. Considering its safety and good patient compliance, it is a simple adjunct to conventional anti-*H. pylori* antibiotic strategies [72].

It should be noted that rather than introducing new organisms to the gastrointestinal microbiota, postbiotics modulate indigenous probiotic strains in patients, demonstrating their supportive role in the preservation of beneficial microbiota, the formation of eubiosis conditions and the stabilization of host homeostasis [2].

#### 3.2.2. Fortify the Epithelial Barrier

Certain postbiotics enhance mucosal barrier function through the alteration of secreted proteins. When administrating the active and heat-killed *L. rhamnosus* to mice with colitis, protection against the rise in mucosal permeability and restoration of barrier function can be observed, which may be attributed to the upregulation of myosin light-chain kinase and zonula occludens-1 in intestinal epithelial cells [73]. Synergism of mucosal protectors and postbiotics has been verified in intestinal cell models. The same combination, which resulted in an increase in transepithelial electrical resistance (TEER) and a decrease in paracellular flux, was also evaluated in CacoGoblet^®^ cells that had been exposed to *E. coli* [74]. Moreover, short-chain fatty acids (SCFAs) modulate the trans-permeability in Caco-2 cells through similar mechanisms, enhancing TEER values and the expression of tight junction protein genes [75,76].

Purified EPS from lactic acid bacteria and bifidobacteria demonstrated the defense against infections in several previous research papers [62,77]. According to some publications, the antibacterial properties of EPS-containing postbiotics may be connected to the formation of a protective biofilm that protects the host epithelium from pathogens or their toxins [77].

A growing body of research demonstrates that certain Gram-positive bacteria products activate signaling pathways, such as TLR2, which results in anti-inflammatory states and plays a critical role in boosting transepithelial resistance to bacterial invasion [2,78].

#### 3.2.3. Modulation of Immune Responses

According to research by Tejada-Simon and Pestka, probiotic bacteria’s whole inactivated cells, cell components, as well as cytoplasmic fractions activate macrophages to produce cytokines and nitric oxide, thus indicating that bioactive substances may be present throughout the probiotic cells [79]. Here we discuss the mechanism of the postbiotics regarding the modulation of immune responses in three dimensions: whole inactivated cells, bacterial components and metabolites.

Certain whole-cell postbiotic products of *Lactobacillus* have demonstrated the anti-inflammatory (downregulation of IL-6, TNF- α and upregulation of IL-10) and anti-oxidative (removal of free radicals) properties in vitro and in vivo experimental animal models [80]. ILs are immune-glycoproteins and are involved in inflammatory responses by modulating multiple growths and the activation progress of immune cells [81]. For example, heat-treated *Bifidobacterium longum* as a whole-cell postbiotic has demonstrated various barrier protection properties, such as antioxidation, anti-inflammation, and the inhibition of bacterial colonization [82].

With the exception of external bacterial products, the structural elements, especially the cell envelope, which is the outermost structure that immune system cells initially interact with, should play a significant role in mediating immunomodulatory activity. In immunomodulation, toll-like receptors (TLRs) are appropriate and the most common targets for ligand-drug discovery strategies, which make postbiotic products possible in inflammatory diseases and autoimmune disorders [83]. Peptidoglycan (PGN) and lipopolysaccharide (LPS) extracted from bacteria have been the subject of several investigations, which have shown that both molecules stimulate the immune system in a receptor-dependent manner. The primary sensors of the innate immune system are specialized conserved pattern recognition receptors (PRRs) on host cell membranes, involving TLRs and the nucleotide-binding domain (NOD) proteins (or NOD-like receptors, NLRs), which recognize PGN and LPS as ligands associated with pathogens [84]. Teichoic acids (TAs) can be covalently bonded to the cytoplasmic membrane or peptidoglycan (wall teichoic acids, WTAs) (lipoteichoic acids, LTAs). It has been suggested that TLR2 is the mechanism by which TAs from *lactobacilli* cause proinflammatory reactions. Additionally, it has been indicated that symbiotic intestinal bacteria and Gram-positive probiotics regulate the immune response to pathogens via their TAs, limiting an excessive inflammatory response [85]. The surface layer (S-layer), consisting of the self-assembly of protein or glycoprotein subunits on the outer surface, allows *lactobacilli* to stimulate the host immune system. In a study conducted by Konstantinov, *L. acidophilus* SlpA was recognized and bound to a C-type lectin receptor existing on both macrophages and dendritic cells [86]. Indeed, *lactobacilli* absent of S-layer proteins showed relatively inadequate adhesion ability to the enterocyte.

Finally, genomic DNA also enables postbiotics to interact with the host immune system. Unmethylated CpG sequences contained in prokaryotic DNA have immunogenicity properties in vitro and in vivo, according to convincing published literature [87]. Researchers from the same period observed that bacterial genomic DNA extracted from pure bifidobacterial cultures of VSL#3 (a probiotic commercial product) affected cytokine production in peripheral blood mononuclear cells (PBMCs), with the tendency towards a low level of IL-1b and a high level of IL-10 [88]. An in vivo mouse investigation also validated the anti-inflammatory properties of genomic DNA from VSL#3, revealing that TLR9 signaling was crucial in mediating such anti-inflammatory response [89].

**Figure 2:**
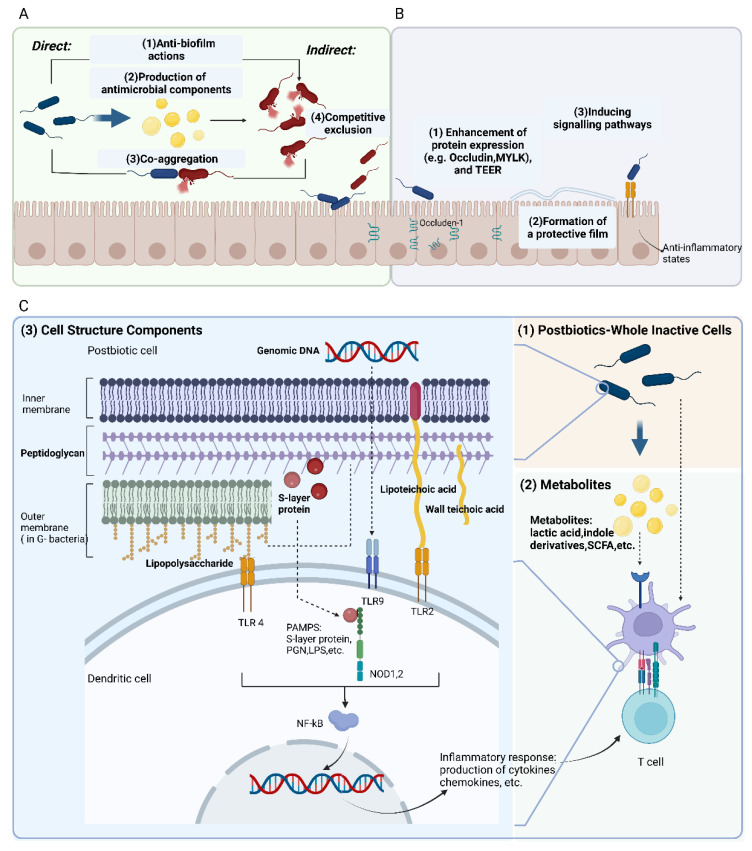
Predominant mechanisms of postbiotic action. (**A**). Protective modulation against pathogens. (**B**). Fortify the epithelial barrier. (**C**). Modulation of immune responses. (figure was created with Biorender. com).

When it comes to metabolites from microorganisms, lactic acid can influence the immune system by, for instance, causing intestinal CX3CR1+ cells to protrude their dendrites in a GPR31-mediated manner [90]. Similarly, indole derivatives secreted by *Limosilactobacillus reuteri* can activate the aryl-hydrocarbon receptor in CD4+ T cells in the intestinal tissue of mice, involving differentiation into CD4+ CD8αα+ intraepithelial lymphocytes [91]. SCFAs with the ability to reduce inflammation and inhibit the growth of malignant cells have been shown to be effective therapeutically in the treatment of inflammatory bowel disease (IBD) and colorectal cancer [92]. The findings of a randomized clinical trial indicate the correlation between the increase in the SCFAs viz. acetate and butyrate and the reduction of the pro-inflammatory cytokine IL-15 when providing IBS patients with *L. paracasei* CNCM I-1572 [93]. Based on genetic analysis of associated bacteria, there may be additional immunostimulatory microbial metabolites in postbiotics including histamine and branched chain fatty acids, the effects of which cover a variety of immunological responses, such as the inhibition of NF-B [5] (Figure 2).

### 3.3. Applications of Postbiotics in Different Fields

#### 3.3.1. Applications in the Food Industry

Fermentation is the most prevalent process with applications of postbiotics, and strains of *Lactobacillus* and *Bifidobacterium* are commonly used as producer strains [94]. The dairy industry benefits greatly from the EPS of specific strains of dairy starter cultures because EPS has significant control over the rheological characteristics of fermented dairy products and lowers their moisture content [95]. Moreover, postbiotics from *Lactobacillus plantarum* can exert efficacy as a bio-preservative to extend the shelf life of soybeans [96]. Combining the above two kinds of application, MicroGARD is a commercial preparation made by Danisco that has received FDA approval and is utilized as a premier biopreservative in extensive dairy and food matrices. It is a fermented version of *Propionibacterium freudenreichii* subsp. *Shermanii* found in skim milk [97]. Other novel approach involves increasing vitamin B and decreasing toxic components during probiotic-induced fermentation [94].

#### 3.3.2. Pharmaceutical Applications

Many postbiotics products in the experimental research stage and not being applied to the clinic yet, also show great application potential. Giordani B et al. in 2019 conducted research and discovered that the biosurfactants of *L. gasseri* had antibiofilm capacity against methicil-lin-resistant *S. aureus* (MRSA). Another example is a heat-stabilized *acidophilus* containing medication called Lacteól Fort (Laboratoire du Lacteól du docteur Boucard, France), which has been proven effective in the treatment of acute diarrhea and IBS [2] by randomized controlled trials. At present, industries are intending to place postbiotics into a regular pharmaceutical product matrix because of their stable pharmacodynamic features and beneficial effects in clinical application [98].

#### 3.3.3. Biomedical Applications

Postbiotics enhancing the effects of vaccination in the elderly has been proven by increasingly more evidence, the underlying mechanism of which includes sustainable antibody production and NK-cell activities. Research in 2016 demonstrated that the concentration of antibody to type A/H1N1 and B antigens were improved in an elderly subgroup with heat-killed *Lactobacillus paracasei* jelly [99]. Moreover, similar to parent probiotics, the use of postbiotics is a promising strategy to treat pediatric infectious diseases in under-five-year-olds because it exerts immunomodulatory as well as antimicrobial effects [100]. Postbiotics are also able to serve as a novel strategy for food allergy in pediatrics because of their unique characteristics against parent live cells [101].

Research demonstrates that the microbiome-metabolome axis in the gastrointestinal tract is affected and therefore graft versus host disease colitis can be alleviated through probiotics or postbiotics application [102]. Similarly, the combination of postbiotic butyrate and active vitamin D could be a possible treatment for infectious and autoimmune colitis [103]. Apart from gastrointestinal diseases, extensive research has proven that specific postbiotic metabolites affect the differentiation and function of CD4+ T cells, with results indicating that postbiotics could be a promising perspective to treat allergic rhinitis [104]. Moreover, urinary tract infections (UTIs) should be further explored based on the results suggesting that mucosal protectors might lessen the intestinal reservoirs of uropathogenic *E. coli* strains [105]. Accordingly, some research indicates that metabolites generated by *lactobacilli* (hydrogen peroxide and lactic acid) act in concert to eradicate uropathogenic organisms in vitro [106,107] and could serve as the foundation for the creation of UTI management products with postbiotics.

Furthermore, various postbiotic molecules have attracted interest because of their wide modulation effects in obesity, coronary artery diseases, and oxidative stress through the capacity to trigger the alleviation of inflammation reactions and pathogen adherence to gastrointestinal tract, etc. Presently, postbiotic preparations have also been granted patents as bio-therapeutics for a specific health benefit of “immune-modulation” [108].

### 3.4. Advantages of Postbiotics Compared with Probiotics

The use of non-viable postbiotics as a safer option has gained popularity as safety concerns over the use of live strains have surfaced in certain patient populations, including immunodeficient subjects, infants and vulnerable patients [34,48,51]. They could significantly reduce consumer risk of microbial translocation and infection [109].

Calculating the percentage of dead cells in a probiotic culture that is still viable will be difficult. Therefore, changing percentages of dead cells may be the origin of the variation in responses usually found with living probiotic products. However, it is simple to demonstrate that postbiotics are devoid of any living organisms. Postbiotics-based products would be long-lasting and extremely simple to standardize, making them easier to store, have a longer shelf life and facilitate logistics under extreme environmental conditions. [34,82].

**Figure 3:**
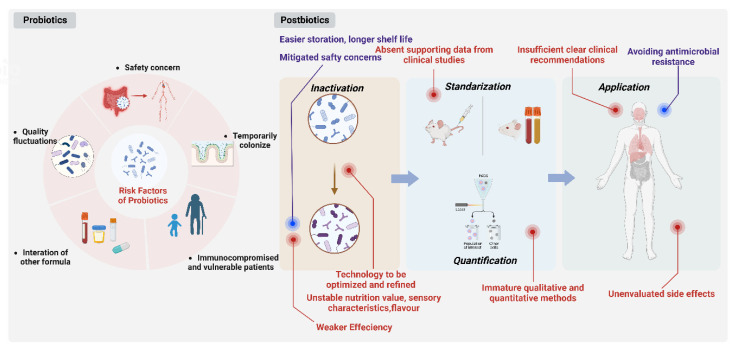
Comparison between probiotics and postbiotics in application.

By lowering the likelihood of the transmission of antibiotic-resistant genes, using inactivated bacteria can have significant advantages. Probiotic use is now discussed in terms of antimicrobial resistance prevention techniques [110,111] and the need to stay away from long-term pharmaceutical treatments and their negative effects [110]. The use of non-viable probiotics as an alternative therapy is increasingly accepted due to the high incidence of antibiotic resistance in live probiotic applications (Figure 3).

### 3.5. Drawbacks of Postbiotics

Postbiotic products have been proven to be a relatively weaker influence on the modulation of intestinal metabolism or gene expression affecting nutrition metabolism when compared with corresponding probiotics. For example, live cells of *Bifidobacterium breve* M-16V displayed enhanced immunomodulation effects in contrast to postbiotics, which is mainly reflected in the inhibition of pro-inflammatory cytokines in spleen cells and more significant alteration of intestinal metabolism [34].

The type of technology in the inactivation process might relate to products with variable functionality in comparison with the progenitor microbial product according to the microbial inactivation degree achieved. For example, it has been demonstrated that different heat treatments ranging from air drying, freeze drying to spray drying can significantly impact the viability and immunomodulatory properties when dehydrating probiotics [112] On the other hand, volatility in the nutritional value, sensory characteristics and flavors caused by traditional thermal processing including pasteurization, tyndallization and autoclaving frequently occurs, thus establishing a reliable controllable range, and the acceptance of the original product audience requires more investigation [5]. Thermal processing, therefore, may not be the best option, especially when a postbiotic product is used as a food supplement. Emerging technologies such as electric field, ultrasonication, high pressure, ionizing radiation, pulsed light, magnetic field heating and plasma technology [113] could possibly be applied to inactivate microorganisms and generate postbiotics to obtain safe and stable foods with retained overall quality and value.

The composition and quantity of a postbiotic product must be described and measured using appropriate methods. These techniques should be accessible for both quality control at the production site, and for a precise product description that enables duplicate research. An emerging technique, flow cytometry, is now gradually substituting traditional technology such as plate counting for microbial counting and enumeration [114].

Inconsistent, vague, and frequently reliant on patient requests, postbiotic product recommendations also suffer from similar issues, according to a recent study on healthcare providers’ probiotic prescription practices. This means that the patient or the pharmacist made the postbiotics choice based merely on their own experience for a significant portion of the time [4]. It is vital to address the insufficient, clear and specific clinical recommendations and the absence of supporting data from clinical research.

In the group receiving the inactivated *L. acidophilus* with micronutrients, side effects appear involving severe to moderate dehydration, abdominal distension, and vomiting ranging from mild to severe [115]. Postbiotic therapies’ safety and potential risks have not been thoroughly researched or understood. To ascertain the effects and safety of various postbiotics, additional multicenter studies are required (Figure 3).

## 4. Future Development

There remains a variety of obstacles that need to be overcome for postbiotics, a new generation of functional foods, in order to achieve stable and beneficial effects through rational design and provide improved protection against infections and other disorders [116]. The Human Microbiome Project has become the research spotlight that scientists are dedicated to and study all over the world as a “second human genome project”, since metagenomic sequencing offers a better understanding of microbiota metabolic activity [117,118]. Generally speaking, a better comprehension of the intricate probiotic-pathogen interactions in the actual human gastrointestinal system will aid in the development of more specialized treatments for various conditions, as well as a better understanding of the degree to which components derived from bacteria are active in vivo [116,119], which leads to a better-defined benefit–risk ratio. At the same time, individualized optimal treatment plans should be formulated based on patients because research has demonstrated that baseline concentrations of immune factors affect the alteration, after consuming a postbiotic product [39].

A precise limit on allowable live microorganisms remaining after postbiotic preparation is needed for regulators. The majority of postbiotic products will contain some survivors depending on the inactivation conditions [2]. Different inactivation technologies and procedures such as heat, high pressure and exposure time to oxygen for anaerobic microorganisms may leave behind varying quantities of viable cells of the progenitor microorganisms [5]. Determining the best conditions for inactivating while maintaining the cell structure is considerable for achievement of the optimal nutritional, physical, rheological, or sensorial properties.

For the next generation products, purification of these components and quantification of their effects will likely enable greater uniformity, culminating in highly specialized and secure products adopted to patient-tailored therapy [4]. At present, flow cytometry is emerging as an alternative to plate counting for microbial detection and enumeration [114] with advantage of high efficiency and being able to separate a microbial population into live, damaged and dead cells [5].

Additionally, it is important to analyze how the in vitro results and animal models relate to the unique features of the human intestine, particularly the colon, which has a stratified layer structure with the gut microbiota predominating in the outer layer [4]. Through the combination of in vitro, in vivo, clinical studies, as well as biochemical evaluations, the more accurate mechanisms of postbiotics can be deeply explored [120].

## 5. Conclusions

Essentially, probiotics are live microorganisms that may proliferate in vivo after administration, leading to incremental efficacy at a specific time period, while postbiotics undergo constant consumption resulting in relatively quicker potency loss. Balancing the safety concerns against performance differences in probiotics and postbiotics, a compromised and optimal prescription is urgent to be proposed when facing a specific patient’s state, which may lie in the combined administration of probiotics and postbiotics in appropriate proportion.

(A) Protective modulation against pathogens. Direct mechanisms: (1) anti-biofilm actions, (2) production of antimicrobial components, (3) co-aggregation; Indirect mechanisms include (4) competitive exclusion. (B) Fortify the epithelial barrier. Three mechanisms are included: (1) enhancement of protein expression, (2) formation of a protective film, (3) inducing signaling pathways. (C) Modulation of immune responses. Three divisions of postbiotic components inducing immune response: (1) whole inactive cells, (2) metabolites, (3) cell structure components in G+ and G− bacteria. (Figure was created with Biorender. com).

The left half demonstrates the disadvantages in probiotics use. In the right half, the drawbacks of postbiotics are shown in red and advantages are shown in blue. (Figure was created with Biorender. com).

## Data Availability

All data generated during this study are included in this published article.
